# Complete uni-port video-assisted thoracoscopic surgery for surgical stabilization of rib fractures: a case report

**DOI:** 10.1186/s13019-023-02167-8

**Published:** 2023-02-06

**Authors:** Zhen Yang, Ming Wen, Weiqing Kong, Xu Li, Zhuan Liu, Xin Liu

**Affiliations:** 1 Department of Thoracic Surgery, The Second Affiliated Hospital of Kunming Medical University, 374th Dianmian Road, Yunnan Province 650101 Kunming, China; 2 Department of Neurosurgery and Thoracic Surgery, The First People’s Hospital of Xuanwei, 131th Jianshe Road, Yunnan Province 655400 Xuanwei, China; 3 Department of Thoracic, Thyroid and Breast Surgery, Panzhou People’s Hospital, 1120th Shengjing Avenue, Panzhou, 553537 Guizhou Province China; 4 Department of Thoracic Surgery, Shanghai Pulmonary Hospital, Tongji University School of Medicine, 507th Zhengmin Road, 200433 Shanghai, China

**Keywords:** Rib fracture, Uni-port, Video-assisted thoracoscopic surgery, Surgical stabilization of rib fractures, Case report

## Abstract

**Background:**

Rib fractures are a common injury in trauma. Potential complications include pain, pneumonia, respiratory failure, disability, and death. Surgical stabilization of rib fractures (SSRF) has become an available treatment option, and complete video-assisted thoracoscopic surgery (VATS) for SSRF is gradually accepted because of minimally invasive and pain relief. To our knowledge, complete uni-port VATS for SSRF has not yet been reported.

**Case presentation:**

A 53-year-old man accidentally fell off a three-meter high scaffolding while working resulting in severe chest pain and shortness of breath. He was found with left 7th through 11th rib fractures with a pulmonary contusion from computed tomography (CT). A 4 cm incision was made in the 7th intercostal space in the midaxillary line, and complete uni-port VATS for SSRF were operated. The patient's pain was significantly relieved after the operation, and the scar was tiny and unapparent.

**Conclusions:**

Complete uni-port VATS for SSRF is a novel and modificatory method of operation with the benefit of minimal invasion, meanwhile, intrathoracic injuries could be treated at the same time. Further study is warranted.

## Background

Chest injuries have become the second leading cause of trauma-related deaths, while rib fracture is the most common type of chest injuries [1]. The potential complications of rib fractures include pain, pneumonia, respiratory failure, disability, and death [2]. Surgical stabilization of rib fractures (SSRF) has become a general treatment option [3], complete video-assisted thoracoscopic surgery(VATS) for SSRF is gradually accepted because of minimally invasive and potential pain relief [4]. In the present case, due to localized refractory pain and displacement of rib fractures, complete uni-port VATS for SSRF was conducted. To our knowledge, complete uni-port VATS for SSRF has not yet been reported.

## Case presentation

**Fig. 1:**
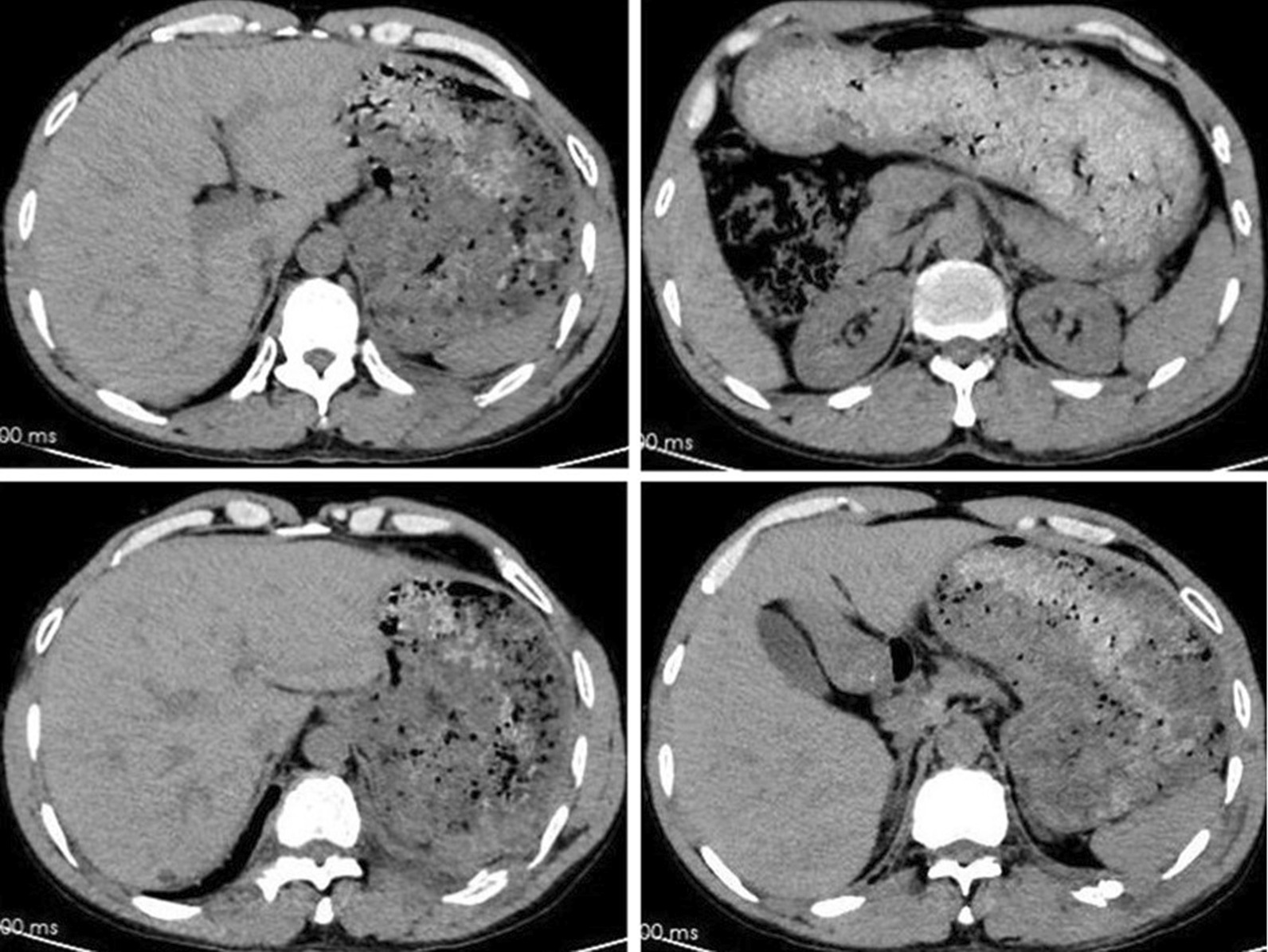
CT of ribs

A 53-year-old man accidentally fell off a three-meter high scaffolding while working resulting in severe chest pain and shortness of breath. Upon arrival at the emergency department of our hospital, physical examination was the following: temperature, 36.8 ℃; blood pressure, 132/86 mmHg; heart rate,101 beats/min; oxygen saturation, 89% (without oxygen inhalation), left chest tenderness, pain on anteroposteria chest compression. Taken chest computed tomography (CT) (Fig. 1), he was found with left 7th through 11th rib fractures with lower left lobe contusion and hemothorax measured about 10% of the chest. After treatment with oxygen, external fixation by band, and pain management, the symptoms were initially relieved, while the pain remained severe several hours later even after the use of pethidine. After consultation, he decided to undergo surgery.

**Fig. 2:**
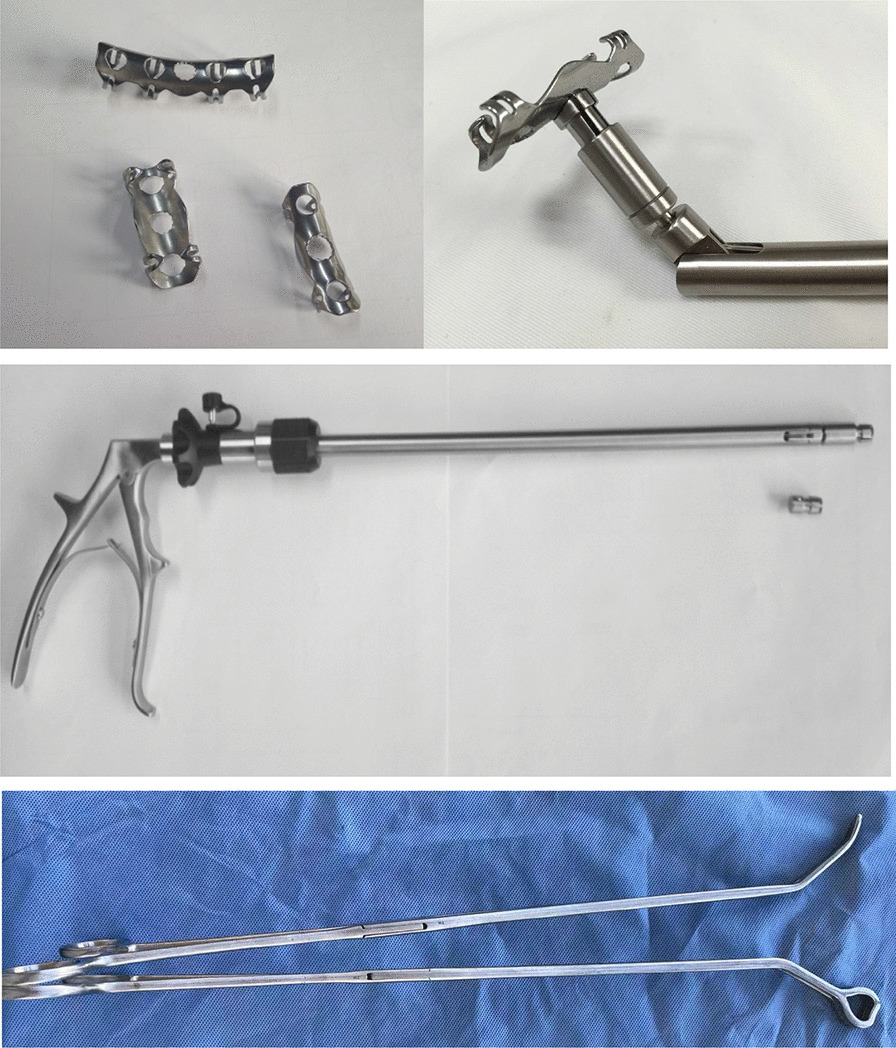
Special instruments

**Fig. 3:**
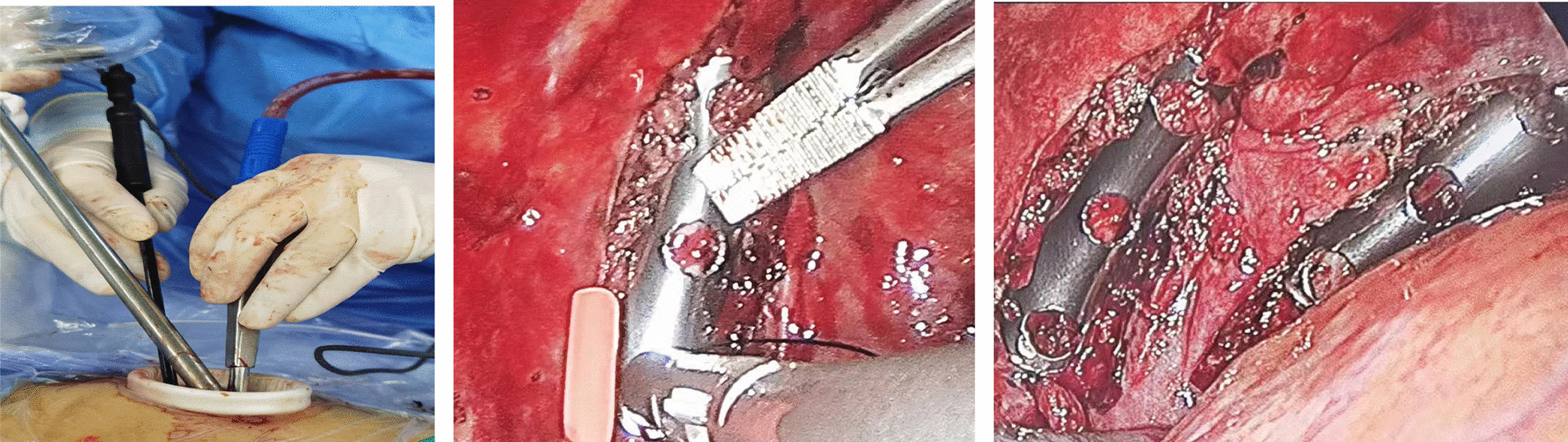
Fixation procedure

**Fig. 4:**
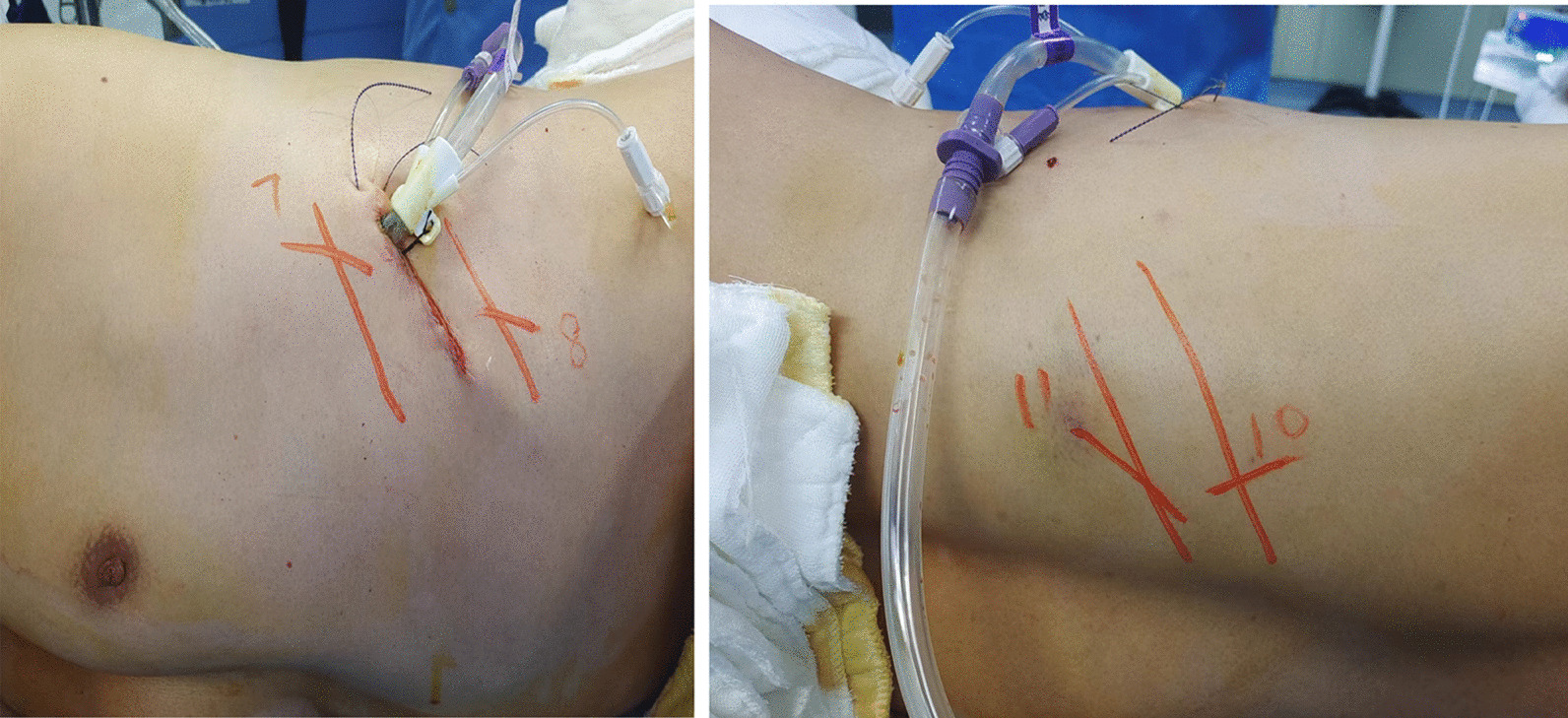
The incision and fractures location

As the left 7th and 8th anterior ribs and the 10th and 11th posterior ribs were dislocated, the patient was taken to the operating room. After the double lumen endotracheal intubation general anesthesia, the patient was placed in the right decubitus position. After skin preparation and draping, a 4 cm incision was made in the 7th intercostal space near the anterior axillary line, where a membrane incision expander was put in, and thoracoscopes and operating instruments operated through the port. Exploration revealed minor active bleeding in the parietal pleura around the fracture, after suction and electrocoagulation, hematoma and deformity were found in the 7th and 8th anterior rib, as well as 10th and 11th ribs, the bone friction sensation around the rib fracture line was evident when pressed. Special instruments were needed (Fig. 2). The rib coaptation boards with 4 or 8 arms (manufactured by Lanzhou Seemine Shape Memory Alloy Co., Ltd, China) were used to fix the fractures. The implantation tool with detachable tong head (manufactured by Lanzhou Seemine Shape Memory Alloy Co., Ltd, China,) was used to connect the rib coaptation board and placed it to the broken ribs. An oval bending forcep (manufactured by Lanzhou Seemine Shape Memory Alloy Co., Ltd, China) was used to reduce displacements. After exposing fractures with an electrocoagulation hook burning, reduction was implement with forcep (for the 10th and 11th rib) or fingers (for the 7th and 8th rib). Loosened the arms of boards under 0℃ ice sterile saline, connected the boards and implantation tool, delivered boards to fractures, and inserted four embracing arms into the upper and lower edges of the fractured rib. After prayed 50℃ sterile saline, boards return to previous shape to clasp and fix the fractured rib. As the result, the rib fractures are stable without screws or wires (Fig. 3). A drainage tube was placed from the incision, and the procedure end up with incision suturing layer by layer (Fig. 4).

**Fig. 5:**
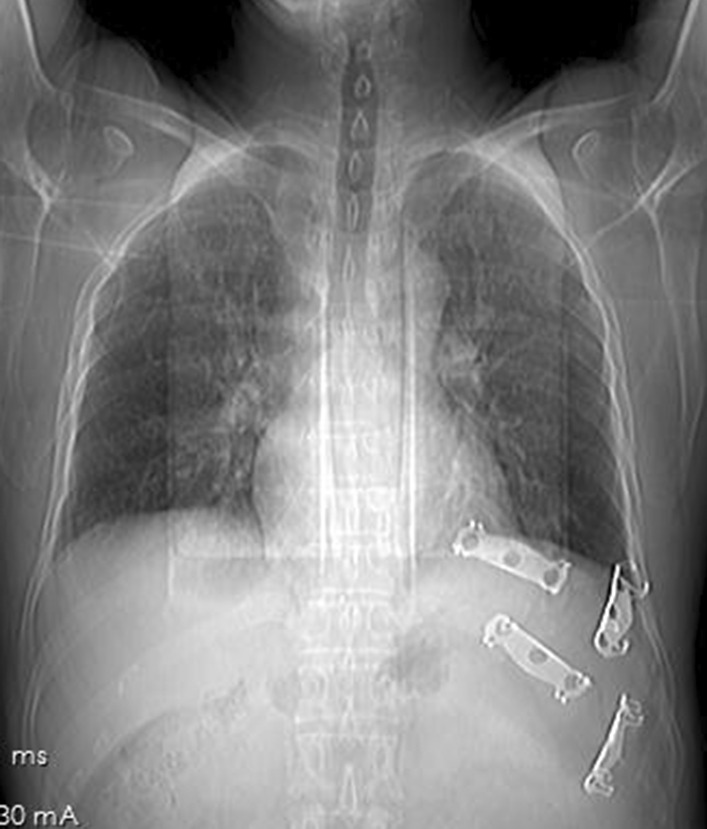
Reexamination of chest CT

Postoperatively, the patient was transferred to the ward for vital signs detection, oxygen inhalation, atomization, pain relief, hemostasis, and fluid therapy. On the post-operative day (POD) 1, he expressed his satisfaction at the apparent ease of the pain, and reexamination of chest CT showed that the fracture was well fixed (Fig. 5). On the POD 2, since the fluid was only 50 ml, the drainage tube was removed, and the patient was discharged next day. A month later, the pain had entirely resolved. The follow-up examination showed the fracture healing well, and the patient returned to work.

## Discussion

Due to many benefits such as relieving pain, increased chest stability, corrected chest wall deformity, improved lung function, shorter ICU stay and ventilator use time, and lower mortality [1, 4, 5], SSRF has become an available option [3].

Flail chest is an universally acceptable surgical indication, however, many relative indications are also existing in clinical practice. A multicenter, prospective, controlled clinical trial indicated that SSRF could obviously reduce the numeric pain score and improved respiratory disability-related quality of life for the patients with nonflail fractures [4]. Some guidelines also pointed the necessity of the surgery for some nonflail patterns, including multiple fractures, marked displacement, localized refractory pain, accompanied with conditions requiring operation [1, 5]. Although most nonflail chest rib fractures can be healed spontaneously after a long time, SSRF could prevents continual friction and promote faster recovery. However, actually, SSRF for nonflail patterns remains controversial due to previous large injuries with surgery. Traditional SSRF requires large incisions to obtain good exposure, which often resulting in enormous associated trauma. The muscles, blood vessels, and nerves in the chest wall were severely damaged, resulting in long-term intractable pain, scar proliferation, upper limb dysfunction, skin paresthesia, and wound infection, which limits the development of SSRF [6]. While VATS can provide a satisfactory visual field in small incisions, reduce complications caused by large incisions, and treat intrathoracic injuries at the same time [6], VATS is gradually accepted and applied in this procedure [7]. We note that there are currently no reports of treatment of multiple nonflail fractures accompanied with hemothorax by uni-port VATS. In our case, we tried to operate with uni-port to alleviate pain, treat the hemothorax at the same time, and minimize the impact on the sensory and motor function to a greater extent. Detailed preoperative planning and appropriate incision selection before the operation were the preconditions for smooth operation. The 7th and 8th anterior ribs and the 10th and 11th posterior ribs needed surgery, and the 7th intercostal incision in the anterior axillary line was selected.. The advantage was that it was directly over the target posterior rib, which was convenient for reducing displacement with forcep and implanting boards to broken ribs. The anterior ribs of the target was located around the incision and manual reduction with fingers could be available. The operation was carried out smoothly, the patient's pain was significantly relieved, and the scar was tiny and not prominent. The postoperative review indicated that the fixation effect was excellent.

The process of uni-port VATS for SSRF was smoothly accomplished. In the future, more surgeries will be performed, then we will conduct a comparative study with multi-port VATS to explore the advantages of uni-port VATS for SSRF.

## Conclusions

Complete uni-port VATS for SSRF is a novel and modificatory method of operation with the benefit of minimal invasion, meanwhile, intrathoracic injuries could be treated at the same time. Further study is warranted.

## Data Availability

The datasets used and/or analyzed in the current article are available from the corresponding author on reasonable request.

## Abbreviations

VATS: Video-assisted thoracoscopic surgery
CT: Computed tomography
SSRF: Surgical stabilization of rib fractures
